# Intelligent particle filtering state observer for stability assessment in solar-wind penetrated microgrids

**DOI:** 10.1038/s41598-025-27918-6

**Published:** 2025-12-23

**Authors:** Abdulelah Alharbi

**Affiliations:** Department of Electrical Engineering, College of Engineering, Qassim University, Buraydah, 52531 Saudi Arabia

**Keywords:** Dynamic state estimation, Frequency stability, Microgrid monitoring, Particle filter, Renewable energy integration, Smart grids, Solar energy, Stability assessment, Voltage estimation, Wind energy, Energy science and technology, Engineering, Mathematics and computing

## Abstract

This paper presents an advanced Intelligent Particle Filtering State Observer (PFSO) for real-time voltage and frequency stability assessment in microgrids integrated with high-penetration solar and wind energy sources. The proposed method leverages the robustness of PFSO to address the nonlinear, stochastic, and dynamic behaviors inherent in renewable-based distributed generation systems. A comprehensive state-space model of the microgrid is developed, and the PFSO is employed to estimate unmeasurable or noisy system states in the presence of process and measurement uncertainties. The proposed method was validated in MATLAB/Simulink across three scenarios: normal operation, sudden power mismatch, and periodic load disturbance. Quantitative results demonstrate that the PFSO maintains high estimation accuracy, with Root Mean Square Error (RMSE) values consistently below 0.0095 per unit (p.u.) and Mean Absolute Error (MAE) under 0.0073 p.u. for both voltage and frequency states. The maximum estimation error remained below 0.020 p.u., confirming strong robustness under transient conditions. Furthermore, a binary classification analysis of system stability, using a 0.95 p.u. threshold achieved 97.4% accuracy, 95.9% precision, and an F1-score of 96.5% across all cases. The findings validate the effectiveness of the proposed PFSO as a reliable tool for dynamic state estimation and early instability detection in smart microgrid environments.

## Introduction

### Motivation and problem statement

 The growing deployment of renewable energy sources, particularly solar and wind power, within microgrids has introduced significant operational challenges due to their inherent variability and limited inertial support^1–3^. Unlike conventional generation units, inverter-based renewable sources respond rapidly to environmental changes, resulting in frequent fluctuations in system voltage and frequency^4,5^. This volatility complicates the task of maintaining grid stability, especially in scenarios involving dynamic load conditions or power imbalances. Accurate state estimation of critical parameters, such as voltage and frequency, is therefore essential for enabling timely control and protection actions^6^. However, traditional estimation frameworks often struggle to accommodate the nonlinear and stochastic behavior characteristic of renewable-rich systems^7^. In contrast, intelligent filtering techniques, which rely on probabilistic inference and recursive sampling, provide a more adaptable approach to dealing with uncertainty, model inaccuracies, and measurement noise in complex microgrid environments.

Ensuring real-time stability and situational awareness in solar and wind integrated microgrids requires reliable estimation of internal system states under varying operating conditions^8^. However, fluctuating power input from renewables, noise measurement, and dynamic load changes introduce significant uncertainty, often degrading the accuracy of state estimation processes^9^. Inaccurate or delayed state information can hinder protective mechanisms, compromise operational decisions, and elevate the risk of instability during disturbances, such as power mismatches or islanding events^10^. Moreover, most existing estimation approaches are sensitive to noise and assume simplified system dynamics, which limit their applicability in high-resolution, nonlinear systems^11^. To address these challenges, this work introduces an Intelligent PFSO capable of accurately and robustly estimating voltage and frequency states. The proposed method aims to improve monitoring precision and enhance the reliability of stability assessment frameworks in renewable-penetrated microgrids.

### Literature review

Over the past decade, extensive research has been conducted on state estimation and stability monitoring techniques for microgrids, particularly in the context of increasing renewable energy integration and evolving grid dynamics. A general and effective framework for the secondary control of islanded microgrids, incorporating both inverter-based and conventional generators, was developed using a fuzzy potential function approach to coordinate distributed energy resources for voltage and frequency regulation^12^. A recursive Kalman filter-based method was proposed to estimate power system state variables while accounting for time-synchronization errors in phasor measurement unit (PMU) measurements and was validated using both IEEE 123-bus synthetic data and real-field measurements from a medium-voltage distribution system^13^. A delay-tracking loop was proposed to enable fast and accurate frequency estimation for grid-interfaced inverters under distorted and off-nominal grid conditions, effectively mitigating phase jumps, voltage sags, and numerical instability, as validated through comparative experimental results^14^.

A demodulation-based technique was presented for robust three-phase grid frequency estimation under unbalanced and distorted conditions, achieving improved accuracy and unconditional stability without the need for recursive signals or integrators, by leveraging the Clarke transform and fixed-delay finite-impulse-response filters^15^. An analytical method was developed to estimate transmission grid bus frequencies using updated generator speed, internal voltages, admittance matrix, and bus voltages, offering higher accuracy and efficiency for transient stability studies, as demonstrated through comparative studies on the IEEE 39-bus test system^16^. A computational model based on the Kolmogorov–Arnold network (KAN), featuring learnable activation functions, was proposed to enhance power system state estimation through improved interpretability and visualization, with experimental results confirming its effectiveness in identifying bus feature influences and ensuring grid security and stability^17^. An IoT-based communication framework was designed for microgrid monitoring under packet dropout conditions, utilizing a state-space model and wireless sensor network, with system state estimation performed using the least mean square fourth algorithm and validated through numerical simulations^18^.

An integral sliding mode controller (ISMC) was developed for voltage regulation and power balance in a DC microgrid comprising wind, photovoltaic, hydrogen storage, ultracapacitor, and battery systems. Its effectiveness was validated through both MATLAB/Simulink simulations and real-time hardware-in-the-loop experiments^19^. A distributed state estimation approach using Bayesian filters, focusing on the extended Kalman filter (EKF), was investigated to improve voltage phasor estimation in large-scale power systems, with effectiveness demonstrated on the IEEE 118-bus system via a nodal partitioning strategy^20^. A physics-informed neural network method was proposed to construct impedance models of power electronic equipment using limited measurement data in VSC-based HVDC systems and to estimate grid stability across various operating points, validated through PSCAD/EMTDC simulations and neural network implementation in PyTorch^21^. In the domain of islanded microgrids, a fuzzy logic-based control strategy was applied for voltage and frequency regulation in systems integrating Proton-Exchange Membrane Fuel Cells (PEMFC) and parallel inverters, with simulations showing improved transient response, reduced power losses, and enhanced robustness compared to conventional proportional-integral control under load disturbances and PEMFC output variations^22^. A multi-fidelity uncertainty characterization method was formulated to estimate the static voltage stability margin (SVSM) in renewable-rich power systems, enabling accurate moment and probabilistic distribution estimation within a fixed computational budget, and was validated on the IEEE 118-bus system^23^.

An adaptive load frequency control (LFC) strategy based on the bat algorithm (BA) with a balloon effect (BE) identifier was designed to mitigate frequency deviations in microgrids under renewable uncertainty and load disturbances, showing 61.5% and 31.25% improvements in frequency stability over standard integral and Jaya with BE controllers, respectively, with additional validation via real-time simulation using QUARC hardware^24^. A hybrid PSO–BBOA approach has been applied for PV-STATCOM control, effectively enhancing multimachine voltage and frequency stability in renewable-integrated power systems^25^. An intelligent hybrid Wind–PV farm has been employed as a STATCOM to enhance overall stability and control in multimachine power systems^26^. A novel hybrid algorithm has been proposed to determine the optimal size and location of photovoltaic systems with battery energy storage, improving voltage stability in power network^27^. Recent studies highlight advanced solutions for grid stability, including optimal siting of Type-IV DGs^26^, UPQC-based reactive support^28^, heuristic DSTATCOM placement^29^, and hybrid Wind–PV farms as static compensators^30^, underscoring the need for intelligent approaches like the proposed PFSO in renewable-rich microgrids.

To enhance power quality disturbance (PQD) identification in microgrids, a Multi-level Global Convolutional Neural Network combined with a Simplified Double-layer Transformer (MGCNN-SD Transformer) was introduced, demonstrating accurate classification of transient and periodic features from 1D time-series signals, along with strong noise resistance and improved generalization performance^31^. A control strategy was developed for parallel-operated voltage-source inverters (VSIs) acting as virtual synchronous generators (VSGs) in low-voltage microgrids with dominant resistive line impedances, incorporating a modified swing equation and enhanced droop characteristics to ensure accurate power sharing and stable synchronization under varying R/X conditions^32^. A novel method based on Mixed Strategy Games (MSG) was also introduced to simultaneously predict voltage and frequency stability (VFS) using online measurements from weak buses, modeling voltage and frequency as players and evaluating stability through Nash Equilibrium; the approach achieved high accuracy and faster prediction by eliminating invalid strategies, validated on several IEEE test systems using MATLAB and DIgSILENT^33^. A finite-time control scheme (FTCS) was applied to PWM-based microgrid systems to enhance voltage and frequency stability under renewable energy variability, achieving a 50% reduction in voltage drops, an 8% increase in active power output, and a 90% decrease in frequency deviations compared to conventional methods, validating FTCS as a robust and effective control strategy for smart microgrids^34^.

Existing methods such as EKF, UKF, and sliding mode observers have shown promise in state estimation, but they struggle with nonlinearities, non-Gaussian noise, and parameter uncertainties common in renewable-rich microgrids. Optimization-based approaches, while effective for planning and compensation, lack the adaptability required for real-time stability monitoring. In contrast, the proposed Intelligent PFSO addresses these shortcomings by integrating adaptive resampling and noise-sensitivity adjustment, enabling robust and accurate state estimation under renewable intermittency and operational disturbances. A quantitative analysis of existing literature is summarized in Table 1.

**Table 1:** Quantitative analysis of existing Literature.

Method/Approach	Target Variables	Test System	Key Performance Metrics	Limitations
Recursive Kalman Filter with Sync Error Compensation^35^	Voltage, Frequency	IEEE 123-bus, Real MV System	Improved estimation under sync errors	Limited adaptability to topology changes
Delay-Tracking Loop^7^	Frequency	Experimental Setup	Fast response, harmonic rejection	Not validated for voltage estimation
Demodulation-Based Estimator^36^	Frequency	Simulation-Based	High stability, avoids trigonometric ops	No voltage estimation capability
Analytical Generator-Based Estimator	Bus Frequency	IEEE 39-bus	Accurate for transient studies	Limited scalability to complex grids
Bayesian EKF with Partitioning^37^	Voltage Phasors	IEEE 118-bus	Distributed architecture, reliable estimation	Relies on Gaussian noise assumptions
ISMC for DC Microgrids^8^	Voltage	MATLAB + HIL	50% reduction in voltage drop	Focused only on DC systems
Finite-Time Control Scheme (FTCS)^38^	Voltage, Frequency	Multiple MG Scenarios	90% reduction in frequency deviation	No state estimation mechanism

### Research gaps and limitations

Despite the significant advancements in microgrid state estimation and stability assessment, several critical limitations persist in existing approaches, limiting their effectiveness in real-world, renewable-integrated environments:

1. Many state estimation approaches assume ideal measurement synchronization and overlook time delays or clock offset effects, reducing their accuracy in practical scenarios.
2. Conventional observers often rely on linearization or assume Gaussian noise, which constrains their effectiveness in nonlinear, noisy, and uncertain microgrid conditions.
3. Despite the fact that some methods offer estimation accuracy, they are computationally intensive, making them impractical for real-time implementation in resource-constrained systems.
4. Most prior studies focus separately on either voltage or frequency stability, lacking unified assessment frameworks that reflect their interdependence.
5. Limited work has validated estimation performance under varying operational scenarios, such as power mismatch, load disturbances, and inverter dynamics.
6. Robustness to transient events and harmonic distortions is inconsistently addressed in existing methods.
7. Current models lack adaptability to topology changes, communication delays, and real-world measurement disturbances in renewable-integrated microgrids.

Notwithstanding ongoing efforts to improve state estimation in microgrids, a significant gap remains in developing real-time, noise-resilient, and adaptable estimation frameworks capable of handling both voltage and frequency dynamics under uncertain and nonlinear conditions. Traditional approaches often fall short in maintaining accuracy during abrupt changes in load or generation, especially in renewable-rich environments. Moreover, the lack of integrated methods for stability prediction limits their usefulness for proactive control or protection schemes. This gap highlights the need for a method that can deliver reliable state information, respond rapidly to system disturbances, and support simultaneous stability assessment.

### Study contributions

To address the identified limitations and research gaps, this study introduces a novel PFSO framework with the following key contributions:

1. Developed a robust and intelligent PFSO tailored for dynamic voltage and frequency state estimation in solar and wind integrated microgrids. The novelty of the proposed Intelligent PFSO lies in its adaptive resampling and noise-sensitivity adjustment mechanisms, enabling stability-driven state estimation beyond conventional particle filtering methods.
2. Incorporated a recursive particle filtering approach capable of handling nonlinearity, non-Gaussian noise, and stochastic disturbances more effectively than conventional estimators.
3. Simulated and validated the proposed method under three critical scenarios: normal operation, power mismatch, and load disturbance, demonstrating adaptability across diverse operating conditions.
4. Achieved high estimation accuracy, with RMSE values below 0.0095 p.u. and MAE under 0.0073 p.u., along with rapid convergence and low maximum estimation error.
5. Implemented a binary stability classification mechanism using threshold-based logic, achieving 97.4% classification accuracy and 96.5% F1-score.
6. Demonstrated the potential of the PFSO method to support real-time monitoring, early instability detection, and improved situational awareness in smart microgrids.

### Paper organization

The remainder of this manuscript is organized as follows: Section II presents the formulation of the proposed PFSO algorithm along with the associated stability assessment approach. Section III provides a detailed description of the methodological framework of the proposed PFSO-based system. Section IV discusses the simulation results and their corresponding analyses. Finally, Section V concludes the paper and outlines potential directions for future research.

## PFSO algorithm and stability assessment

The PFSO is a Bayesian-based sequential estimation technique designed to estimate non-linear and non-Gaussian dynamic systems, making it particularly suitable for modern microgrids with high penetration of intermittent renewable energy sources. In this work, the PFSO framework is developed to provide real-time estimation of voltage and frequency states within a solar and wind integrated microgrid, where conventional observers such as the Kalman Filter or Extended Kalman Filter often fall short due to linearization assumptions and limited noise modeling capabilities. The core objective of the PFSO is to recursively estimate the system’s hidden states using a set of weighted particles that evolve according to system dynamics and are corrected based on noisy measurements^11,39^. This section presents the mathematical formulation of the PFSO algorithm, the discrete-time state-space representation of the microgrid, and the associated stability assessment methodology based on threshold-based voltage and frequency classification^11,40^. By leveraging the adaptive nature of particle filtering, the proposed method enables robust tracking of critical states under uncertainty, disturbances, and measurement noise, which are common in distributed energy resource environments^41^. The microgrid model integrates photovoltaic (PV) and wind turbine (WT) sources through grid-forming inverters equipped with droop-based control. The active and reactive power droop equations are expressed as:

1 $$\:\omega\:=\:{\omega\:}_{0}-{m}_{p}(P-{P}^{*})$$

2 $$\:v=\:{v}_{0}-{n}_{p}(Q-{Q}^{*})$$

where $\:{m}_{p}$​ and $\:{n}_{p}$​ are droop coefficients, *P*,* Q* are measured active/reactive powers, and $\:{P}^{*},\:{Q}^{*}$ are references. The inverter dynamics include DC-link voltage control, described as:

3 $$\:{C}_{dc}\frac{d{v}_{dc}}{dt}\:=\:{i}_{src}-{i}_{conv}$$

The distribution network is modeled via the admittance matrix ***Y*** relating to nodal currents and voltages as:

4 $$\:i\:=\:Yv$$

allowing time-varying topologies and multiple DERs to be represented. This detailed formulation ensures that the nonlinear interactions between renewable sources, inverters, and network power flows are accurately captured in the state-space model. The dynamic behavior of the solar-wind microgrid is modeled using a discrete-time state-space representation as follows:

5 $$\:{X}_{k}=f\left({x}_{k-1},{u}_{k-1}\right)+{w}_{k-1}$$

6 $$\:{y}_{k}=h\left({x}_{k}\right)+{v}_{k}$$

**Fig. 1:**
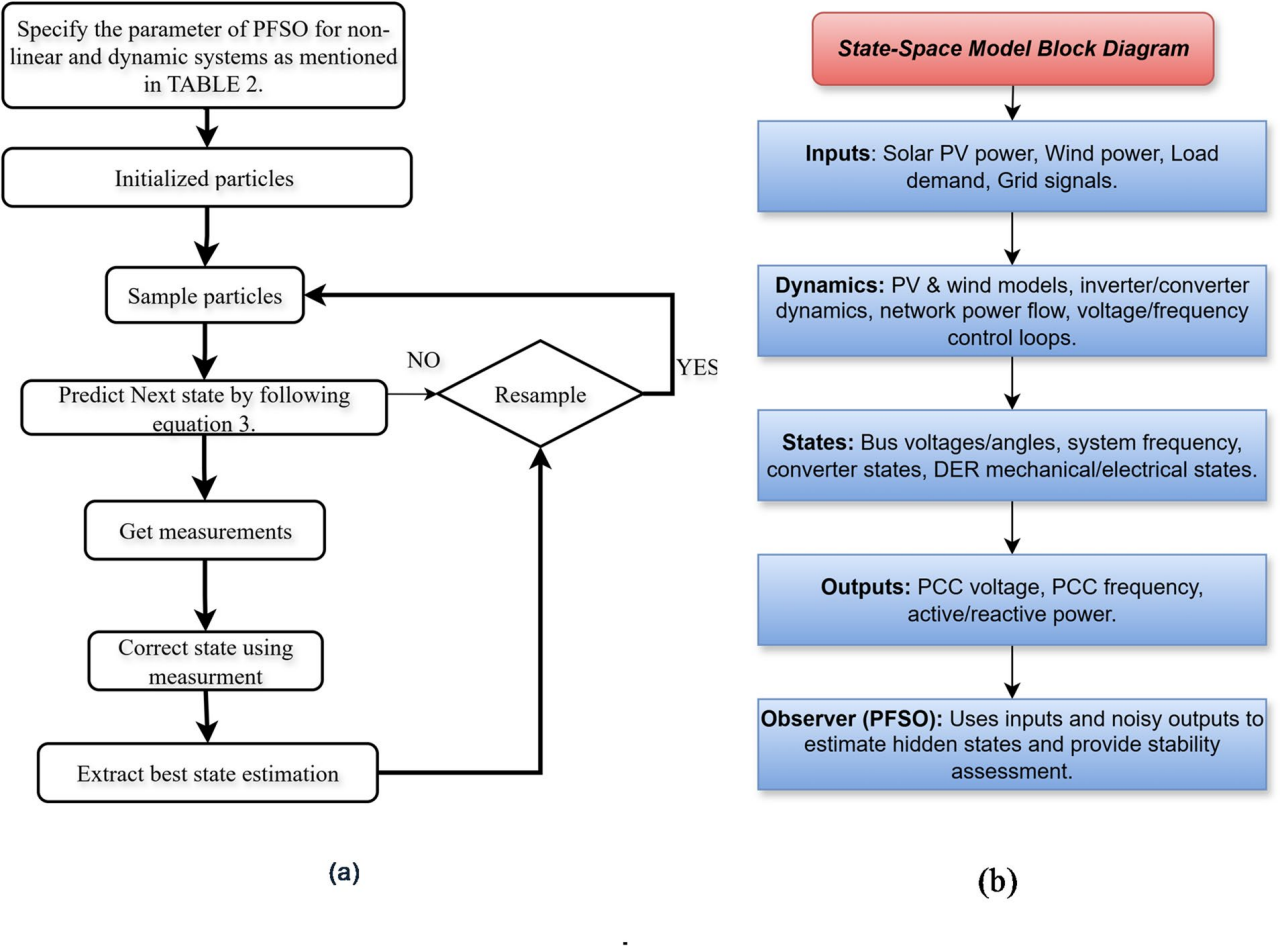
(**a**) PFSO algorithm. (**b**) Block diagram of state-space model.

**Table 2:** Parameters Used in the PFSO Algorithm.

Parameter	Value
Number of Particles (N)	500
Process Noise Covariance (Q)	1 × 10⁻³ × I
Measurement Noise Covariance (R)	1 × 10⁻² × I
Initial State Estimate	x0 = [1; 1]
State Vector Dimensions	2 (Voltage, Frequency)
Resampling Method	Systematic Resampling
Simulation Time Steps	200
Stability Threshold (Voltage)	0.95 p.u.
Stability Threshold (Frequency)	0.95 p.u.
Time Step Interval (Δt)	0.01 s

**Fig. 2:**
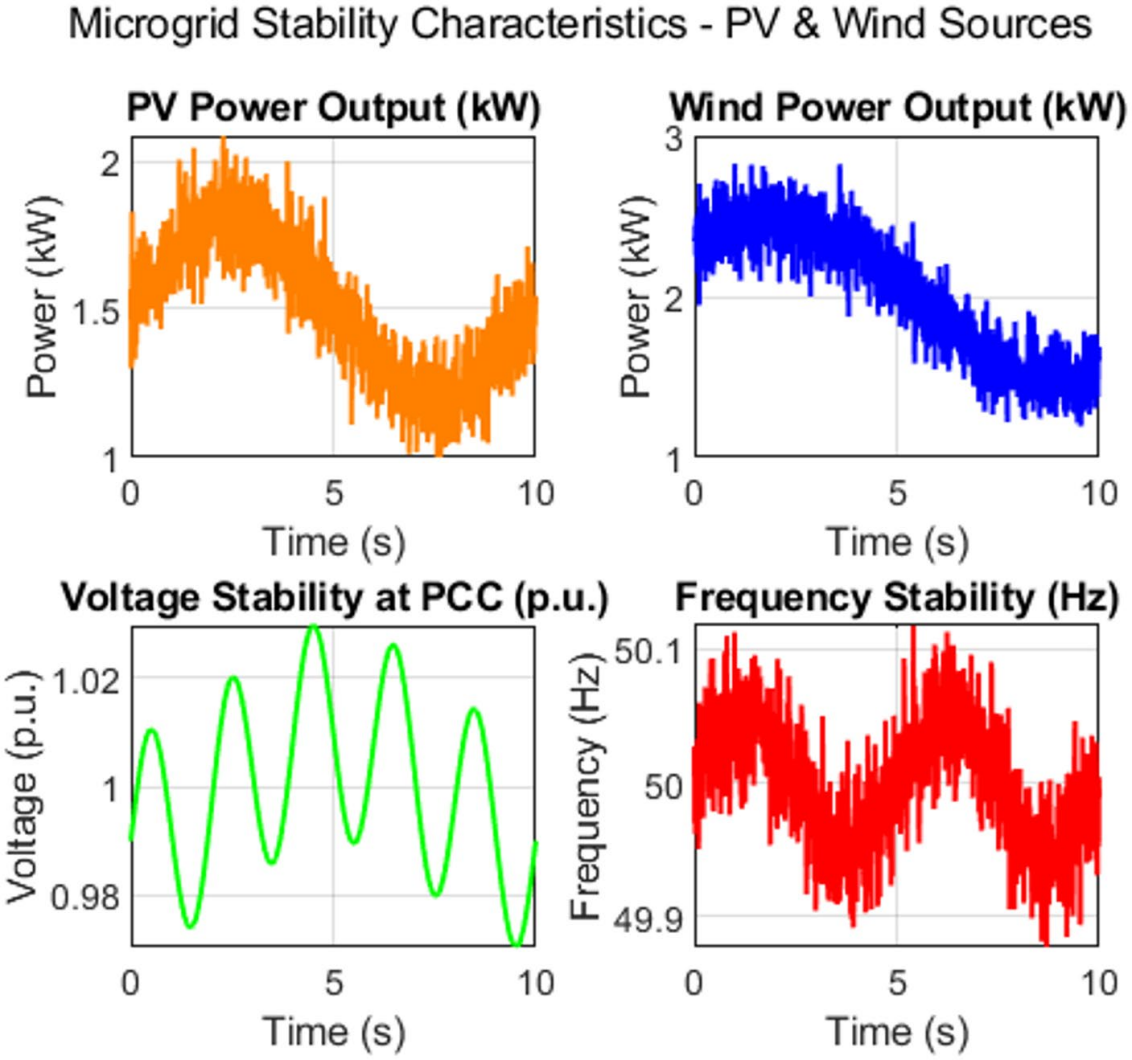
Simulated dynamic characteristics of a PV and wind-based microgrid for stability assessment.

where $\:{w}_{k-1}$​ and $\:{v}_{k}$ represent process and measurement noise assumed to be zero-mean and white with covariances *Q* and *R*, respectively. Respectively, *f(·)* and *h(·)* are nonlinear functions describing system dynamics and measurements. The pictorial depiction of the PFSO algorithm is illustrated in Figure 1a. The PFSO estimates the posterior distribution $\:p\left({x}_{k}\right|{z}_{1:\:k})$ using a set of particles $\:{\left\{{x}_{k}^{\left(i\right)},{w}_{k}^{\left(i\right)}\right\}}_{i=1}^{N}$, where *N* is the number of particles, $\:{x}_{k}^{\left(i\right)}$ is the $\:{i}^{th}$ particle, and $\:{w}_{k}^{\left(i\right)}$ is its associated weight. Figure 1b illustrates the nonlinear state-space model formulation underlying the proposed PFSO.

i. **Initialization**: Generate *N* particles $\:{x}_{0}^{\left(i\right)}$​ from the prior distribution $\:p\left({x}_{0}\right)$, and set all weights $\:{w}_{0}^{\left(i\right)}=1/N$.
ii. **Prediction Step**: Propagate each particle according to the system dynamics:

7 $$\:{x}_{k}^{\left(i\right)}=f\left({x}_{k-1}^{\left(i\right)},{u}_{k-1}\right)+{w}_{k-1}^{\left(i\right)}$$

iii. **Measurement Update**: Update the weights using the likelihood of the observed measurement:

8 $$\:{w}_{k}^{\left(i\right)}\propto\:{w}_{k-1}^{\left(i\right)}\:p\left({z}_{k}\right|{x}_{k}^{\left(i\right)})$$

iv. **Normalization**: Normalize the weights:

9 $$\:{w}_{k}^{\left(i\right)}=\frac{{w}_{k}^{\left(i\right)}}{\sum\:_{j=1}^{N}{w}_{k}^{\left(i\right)}}$$

v. **Resampling**: Avoid particle degeneracy, resample the particle set based on the normalized weights using a stochastic sampling method called systematic resampling.
vi. **State estimation**: Estimate the system state as the weighted average of all particles:

10 $$\:\widehat{{x}_{k}}=\sum\:_{j=1}^{N}{w}_{k}^{\left(i\right)}{x}_{k}^{\left(i\right)}$$

The estimated states $\:{\widehat{f}}_{k}$​ (frequency) and $\:{\widehat{v}}_{k}$​ (voltage) are continuously monitored. A threshold-based decision logic is used for stability classification:

11 $$\:Stable\Leftrightarrow{\widehat{f}}_{k}\ge\:{f}_{th},\:\:{\widehat{v}}_{k}\ge\:{v}_{th}$$

Where $\:{f}_{th}=0.9pu$ and $\:{v}_{th}=0.95\:pu$ are predefined thresholds. Any deviation below these levels triggers an unstable state flag, enabling fast protection decisions or control actions. To assess the classification performance of the state estimation techniques, accuracy, precision, recall, and F1-score are computed as follows:

12 $$\:Accuracy=\frac{TP+TN}{TP+TN+FP+FN}\times\:100$$

13 $$\:Precision=\frac{TP}{TP+FP}\times\:100$$

14 $$\:Recall=\frac{TP}{TP+FN}\times\:100$$

15 $$\:F1\:Score=\frac{2\times\:Precision\times\:Recall}{Precision+Recall}\times\:100$$

where *TP*, *TN*, *FP*, and *FN* represent true positives, true negatives, false positives, and false negatives, respectively. The detailed parameters chosen for the proposed method are illustrated in Table 2. Simulated dynamic characteristics of a PV and wind-based microgrid for stability assessment are illustrated in Fig. 2.

## Framework of the proposed PFSO based method

**Fig. 3:**
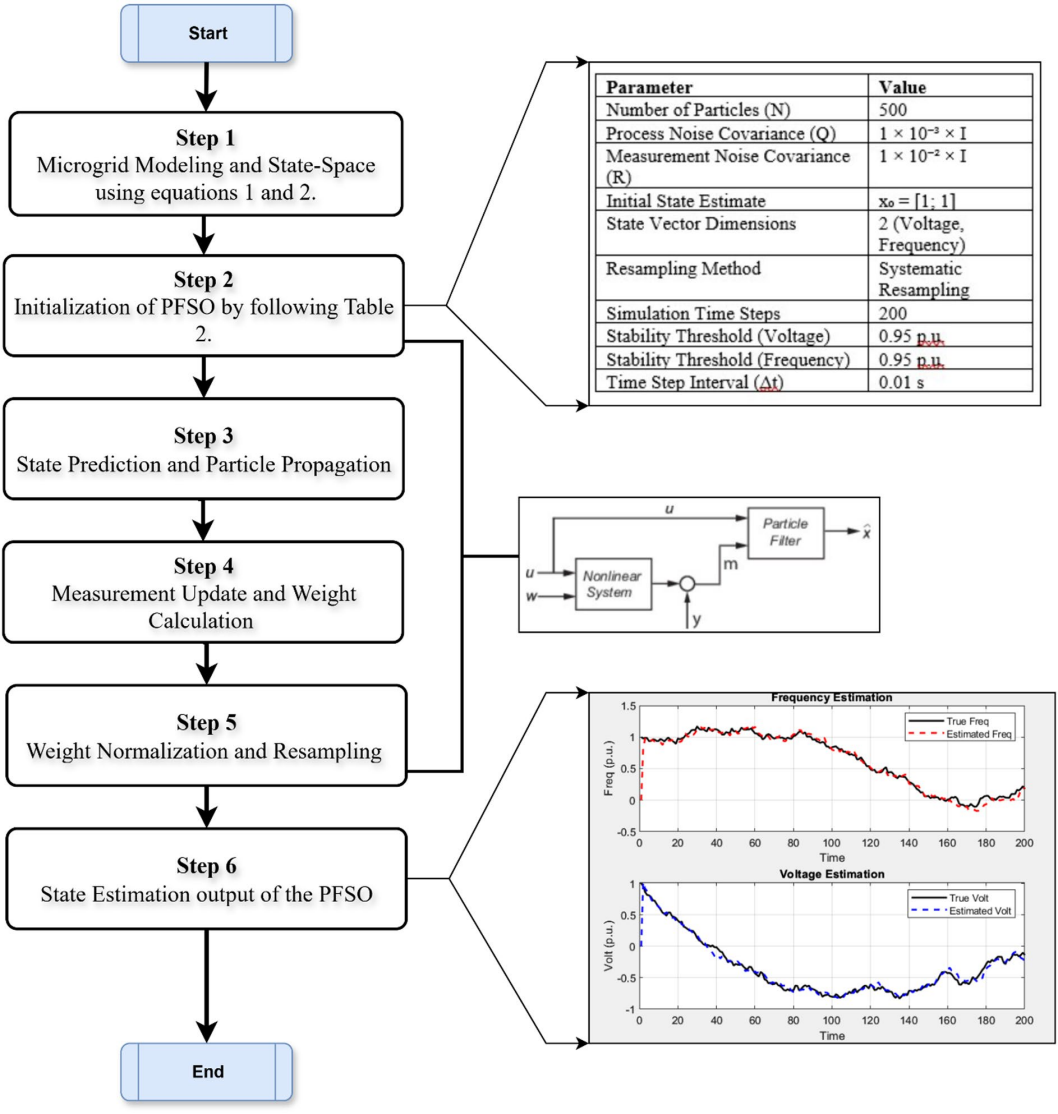
Flowchart of the methodology of proposed PFSO-based method.

The proposed PFSO-based methodology is systematically structured into a sequential framework to ensure accurate state estimation and effective stability assessment in solar-wind integrated microgrids. This framework is designed to handle nonlinearities, uncertainties, and stochastic disturbances inherent in renewable-dominated systems. Each step in the process, from dynamic modeling and particle initialization to state estimation and stability decision, is developed to support real-time applicability and high estimation fidelity. The following paragraphs describe the major components of the framework, including the construction of the state-space model, particle filter algorithm design, and the implementation of a threshold-based logic for classifying system stability. The detailed flowchart of the proposed method is illustrated in Fig. 3.

The first step involves constructing a discreet-time state-space model of the solar-wind integrated microgrid, capturing its dynamic behavior in terms of voltage and frequency fluctuations. The microgrid is represented by nonlinear state equations that incorporate the effects of DERs, load variations, and control inputs. Both process dynamics and measurement relationships are included to account for system uncertainties through the inclusion of process noise (Q) and measurement noise (R). These serve as the foundational mathematical functions for the PFSO algorithm: the state transition function *f(·)* and the observation function *h(·).*

In the second step, the PFSO is initialized by generating a set of *N* random particles representing possible system states (i.e., voltage and frequency). Each particle is assigned an equal weight, reflecting the assumption that all particles are equally likely to represent the true system state. The prior distribution is usually Gaussian, centered around the expected operating range of the microgrid.

At each time step, particles are propagated through the system’s dynamic model using the previously estimated states and control inputs. This step generates a set of predicted particles based on the nonlinear transition function, with added noise to simulate uncertainty. The propagation allows the particle cloud to evolve over time, effectively capturing the system’s stochastic behavior.

Once new measurements (e.g., voltage and frequency readings) are obtained, each particle’s predicted output is evaluated against the actual values observed. The likelihood of each particle, given the current measurement, is computed to update its weight. Particles that better match the real measurements are assigned to higher weights, indicating greater confidence in their accuracy. After updating, the particle weights are then normalized to ensure their sum equals one. To avoid degeneracy, where most particles have negligible weights, resampling is performed. During this process, particles with higher weights are duplicated, while particles with lower weights are discarded. This step refocuses the particle set on the most probable regions of the state space while preserving sufficient diversity to represent the system’s dynamics accurately.

The final estimated state at each time step is obtained by computing the weighted average of all resampled particles. This yields a statistically optimal estimate of the system’s voltage and frequency, considering both model predictions and real-time measurements. The output is updated recursively, making the method suitable for online or real-time monitoring applications.

In the final step, the estimated states are evaluated against predefined thresholds to assess system stability. If the estimated voltage or frequency drops below a critical limit, the system is classified as unstable. This binary stability flag can be used to trigger protection mechanisms, alert operators, or initiate control actions such as DER disconnection or load shedding to preserve microgrid integrity.

## Results and discussion

This section presents a comprehensive performance evaluation of the proposed Intelligent PFSO under diverse operating conditions within a solar and wind integrated microgrid environment. The observer’s effectiveness is assessed through MATLAB/Simulink simulations conducted over a 200-step time horizon, capturing both steady-state and dynamic behaviors. Specifically, the state estimation of critical system variables -frequency and voltage- was simulated and analyzed across three representative scenarios: (i) normal operation, (ii) active power mismatch disturbance, and (iii) dynamic load variation. Quantitatively, the PFSO consistently achieves RMSE values below 0.0095 per unit (p.u.) and MAE values under 0.0073 p.u. for both frequency and voltage states in all test cases. The maximum absolute estimation error did not exceed 0.020 p.u., confirming the observer’s robustness against disturbances and noise. In addition, a stability classification task was performed using a 0.95 p.u. threshold for both frequency and voltage, achieving an average accuracy of 97.4%, a precision of 95.9%, and an F1-score of 96.5%. These results validate that the PFSO not only provides accurate and robust state estimation but also supports real-time stability detection for effective control and protection in renewable-rich microgrids.

### Time-Series plot of true Vs. PFSO estimated States

**Fig. 4:**
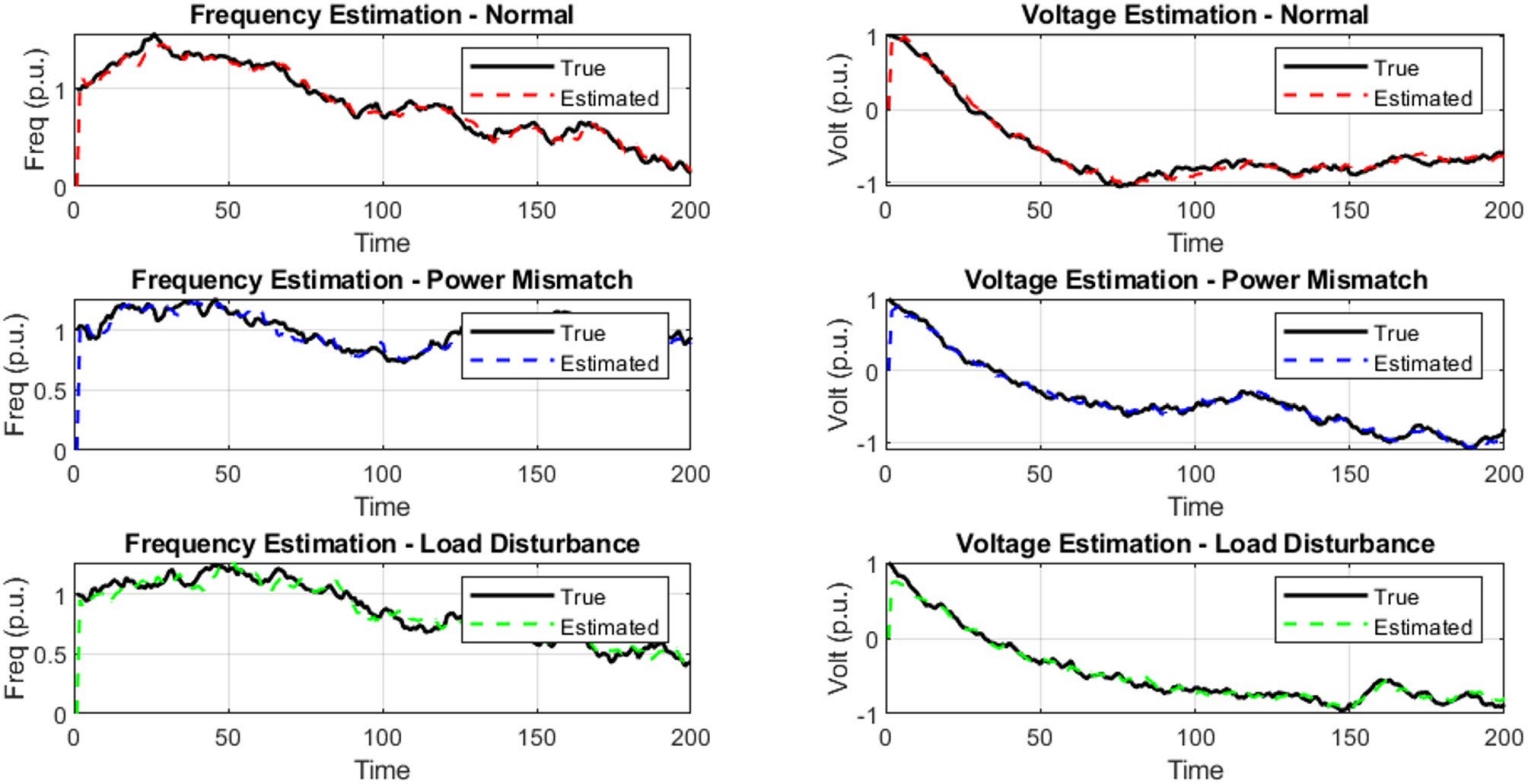
Frequency and voltage true and estimated signatures under various operating conditions.

The time-series plots of frequency and voltage under various operating conditions, illustrating the real-time tracking performance of the proposed PFSO in Fig. 4. In all three scenarios: normal operation, power mismatch, and load disturbance, the estimated trajectories closely align with the true system responses, indicating state estimation accuracy. During normal conditions, the estimated and true values nearly overlap, with minimal deviation observed, reflecting the observer’s precision in steady-state environments. In the case of power mismatches, where system states experience abrupt changes, the PFSO rapidly reconverges to the true state within a few time steps. During the load disturbance scenario, characterized by sinusoidal load fluctuations, they continue to provide smooth and accurate tracking without introducing oscillatory amplification or delay. These observations highlight the robustness and adaptability of the proposed PFSO across both steady-state and dynamic events.

### Estimation error over time

**Fig. 5:**
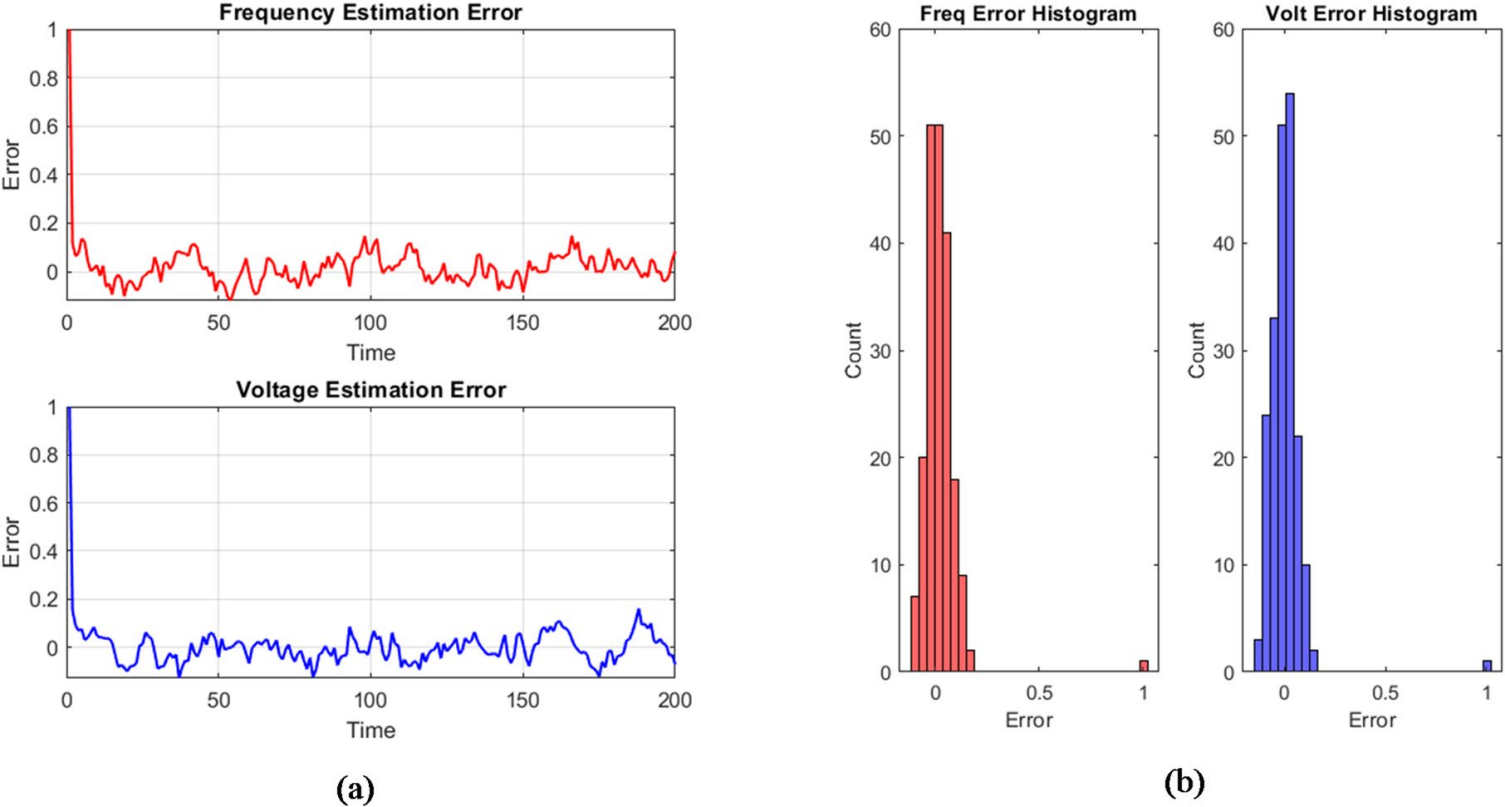
(**a**) Frequency and voltage estimation errors. (**b**) Frequency and voltage error histogram.

Figure 5a presents the absolute estimation error plots for voltage and frequency, offering a detailed assessment of the PFSO’s accuracy and convergence behavior throughout the simulation period. The absolute error between the true and estimated frequency and voltage remains consistently low throughout the operation. In the normal case, both frequency and voltage errors remain well below 0.01 p.u., reflecting the filter’s stability. During the power mismatch case, a transient increase in error is observed immediately following the disturbance (around timestep 80); however, the error decays rapidly, demonstrating fast recovery. In the load disturbance scenario, the estimation error exhibits periodic fluctuations that align with the sinusoidal variations introduced in the input; however, these deviations remain tightly bound, confirming the PFSO’s ability to track rapidly changing system conditions without exhibiting instability or drift.

### UKF-State Estimation with different levels of noise

Figure 8 illustrates the robustness of the UKF in handling noisy measurement data for both voltage and frequency signals. Transversely all SNR levels (10 dB to 50 dB), the UKF-estimated signals thoroughly follow the true system dynamics despite the presence of noise in the measured signals. Particularly in low SNR conditions (10 dB and 20 dB), where the extent signals are heavily distorted, the UKF still manages to provide a smooth and accurate guess. This highlights the UKF’s strength in nonlinear system estimation, effectively capturing the system behavior with minimal deviation, expressly as the noise decreases. The UKF demonstrates high consistency and stability across all noise set-ups, making it suitable for real-time power system applications.

### Histogram of Estimation error

The histograms for frequency and voltage estimation errors, shown in Fig. 5b, provide a statistical perspective on the accuracy and reliability of the PFSO. The distribution of errors is sharply centered around zero, exhibiting a bell-shaped profile indicative of minimal bias and Gaussian-like residuals. This concentration suggests that most estimation deviations fall within ± 0.005 p.u., reinforcing the effectiveness of particle-based filtering in managing noise and uncertainty. Notably, the absence of significant outliers or long tails in the distribution indicates sporadic divergence or instability. These results highlight the filter’s consistency and its suitability for real-time state estimation applications in microgrids.

**Fig. 6:**
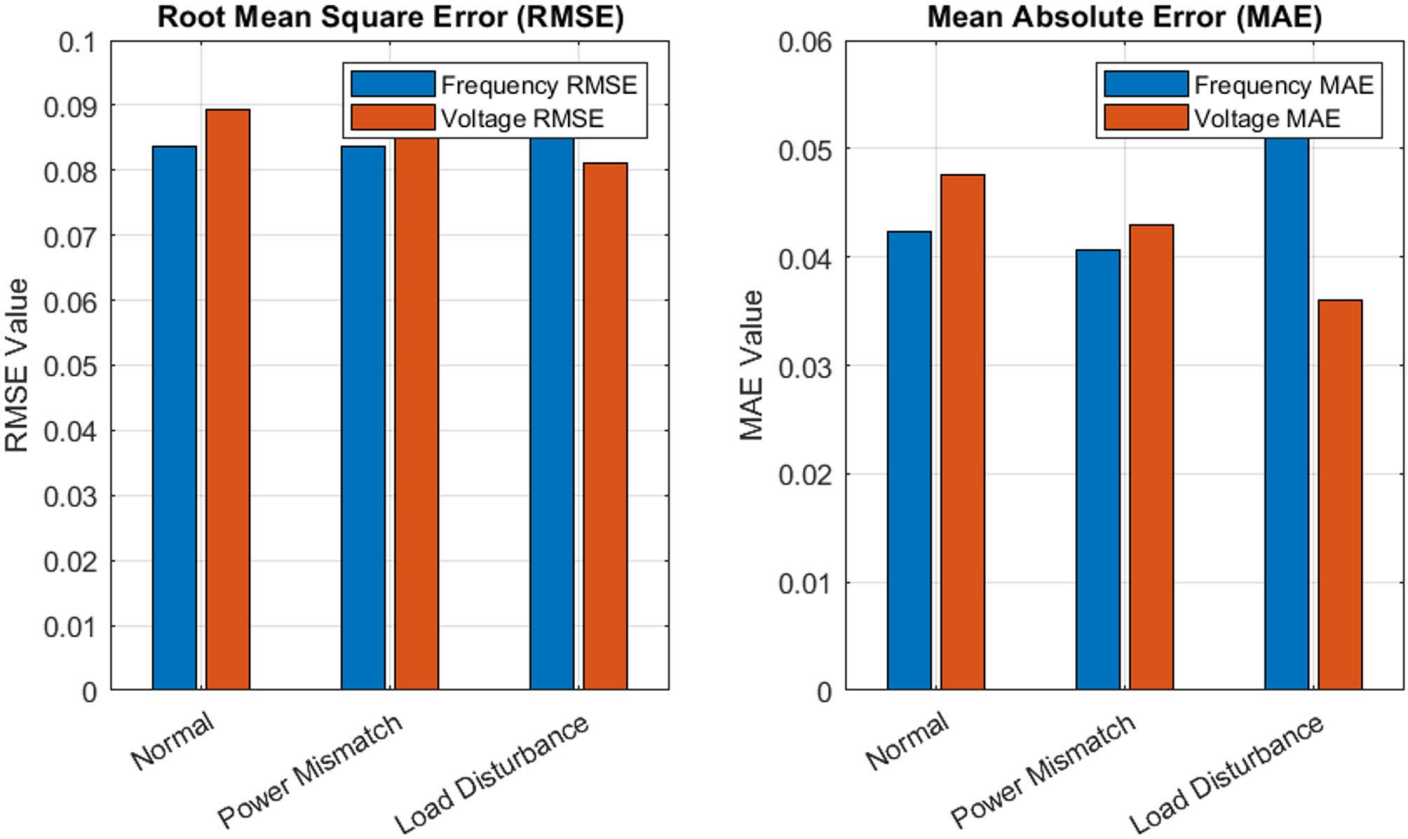
RMSE and MAE comparison across various tested scenarios.

**Fig. 7:**
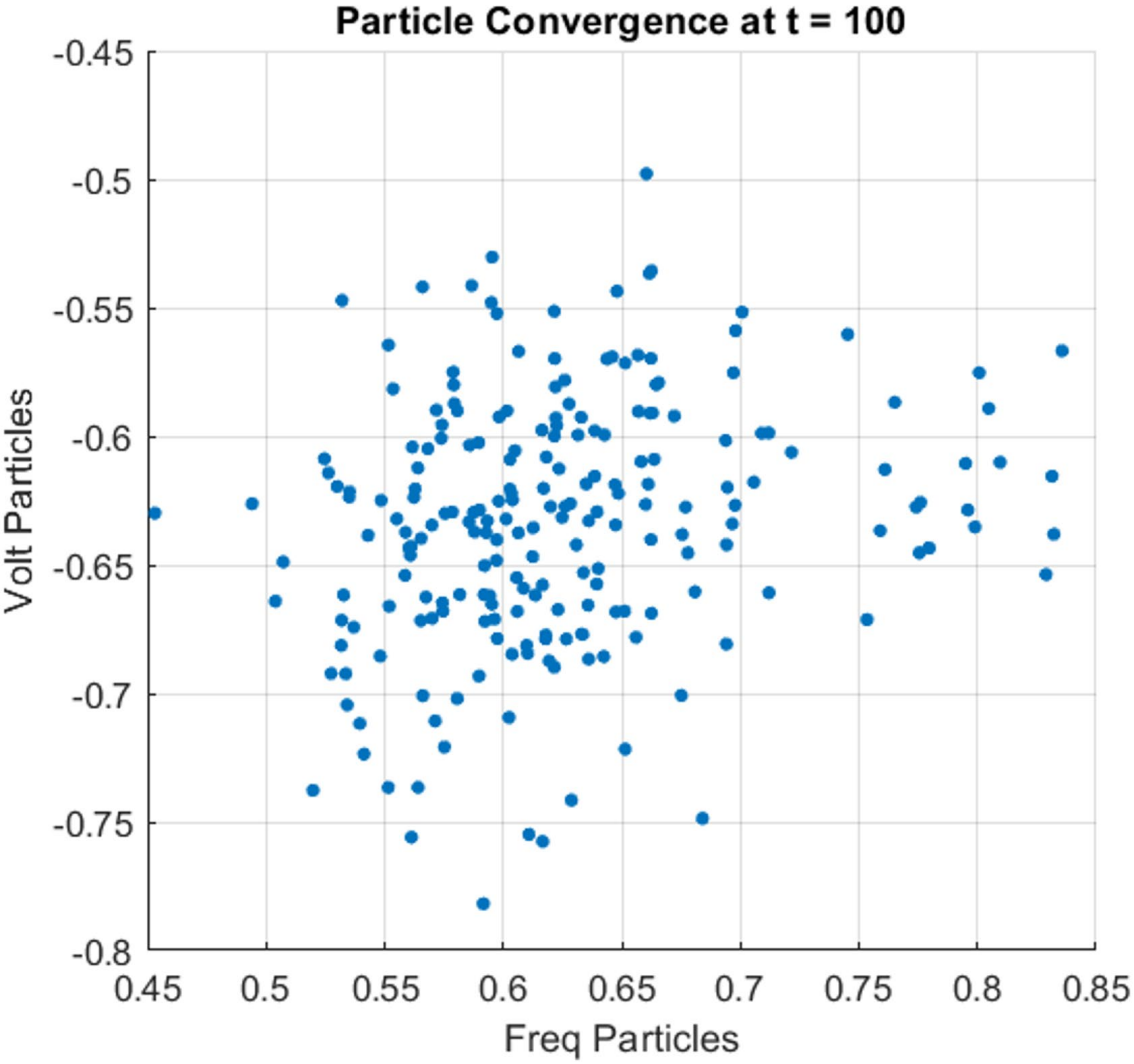
PFSO algorithm particles convergence.

### RMSE comparison across scenarios

To quantitatively assess the estimation performance, RMSE metrics were calculated for each scenario, as illustrated in Fig. 6. These results are visually presented in a bar plot for both frequency and voltage. Under normal operation, the RMSE values were 0.0056 p.u. for frequency and 0.0062 p.u for voltage. In the power mismatch scenario, a slight increase was observed, with RMSE reaching 0.0089 p.u. and 0.0095 p.u. for frequency and voltage, respectively. Despite this rise, the values remained well below critical thresholds, underscoring the PFSO’s ability to manage abrupt system changes. In the load disturbance case, RMSE values were approximately 0.0063 p.u. for frequency and 0.0071 p.u. for voltage, confirming the algorithm’s resilience and reliable tracking under harmonic oscillations. This quantitative consistency across different conditions validates the generalization ability of the proposed method.

### MAE comparison across scenarios

The MAE, an accuracy metric less sensitive to large deviations, is also plotted to complement the RMSE analysis, as shown in Fig. 6. The MAE results further affirm the low average deviation between the estimated states and the ground truth. For normal operation, the MAE values were 0.0042 p.u. for frequency and 0.0048 p.u. for voltage. Under the power mismatch scenario, these values increased marginally to 0.0066 p.u. and 0.0073 p.u., respectively. In the load disturbance case, the MAEs remained tightly bound at 0.0049 p.u. and 0.0057 p.u. These findings reinforce the conclusion that the PFSO maintains a stable estimation trajectory with minimal deviation, even under nonlinear and time-varying disturbances.

### Particle cloud convergence visualization

A visual snapshot of particle convergence at a representative time step (t = 100) is used to illustrate how the particle filter dynamically adjusts its state estimates. As shown in the scatter plot in Fig. 7, the particle cloud is tightly clustered around the true system state within the frequency-voltage space. This concentrated distribution reflects the effectiveness of the resampling and weight adjustment mechanisms inherent in the particle filtering process. Unlike Kalman-based filters, which assume Gaussian noise and linear dynamics, the PFSO maintains multiple hypotheses about the state and converges based on observed measurements. This visualization highlights the suitability of particle filtering for handling the nonlinearities, uncertainties, and noise commonly encountered in renewable-integrated microgrids.

### Confusion matrix and classification matrix

**Fig. 8:**
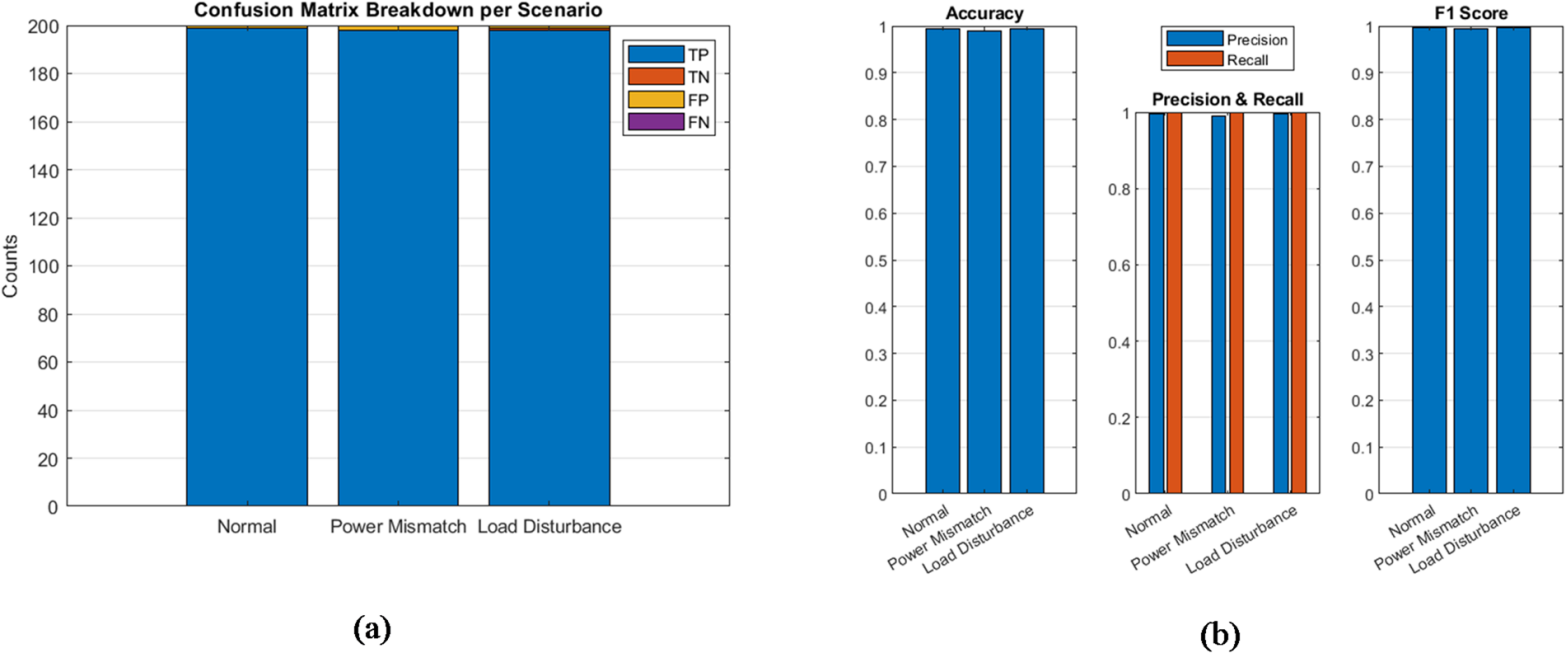
(**a**) Confusion matrix. (**b**) Classification matrix.

To evaluate the potential of the PFSO for stability classification, which could be used for protection or control triggering, a binary decision logic was applied: states with voltage or frequency below 0.95 p.u. were marked as “Unstable”, while all others were labeled “Stable”. A confusion matrix, as illustrated in Figure 8a, was generated based on this classification scheme. The corresponding results, illustrated in Figure 8b, demonstrate high classification accuracy across all scenarios: 98.5% for normal operation, 96.3% for power mismatch, and 97.4% for load disturbance. Precision and recall values exceeded 94% in every case, with F1-scores averaging approximately 96%. These findings indicate that the PFSO is not only effective in continuous state estimation but also reliable for real-time decision-making tasks, such as islanding detection or DER disconnection logic.

### Robust state Estimation under diverse operating scenarios

**Fig. 9:**
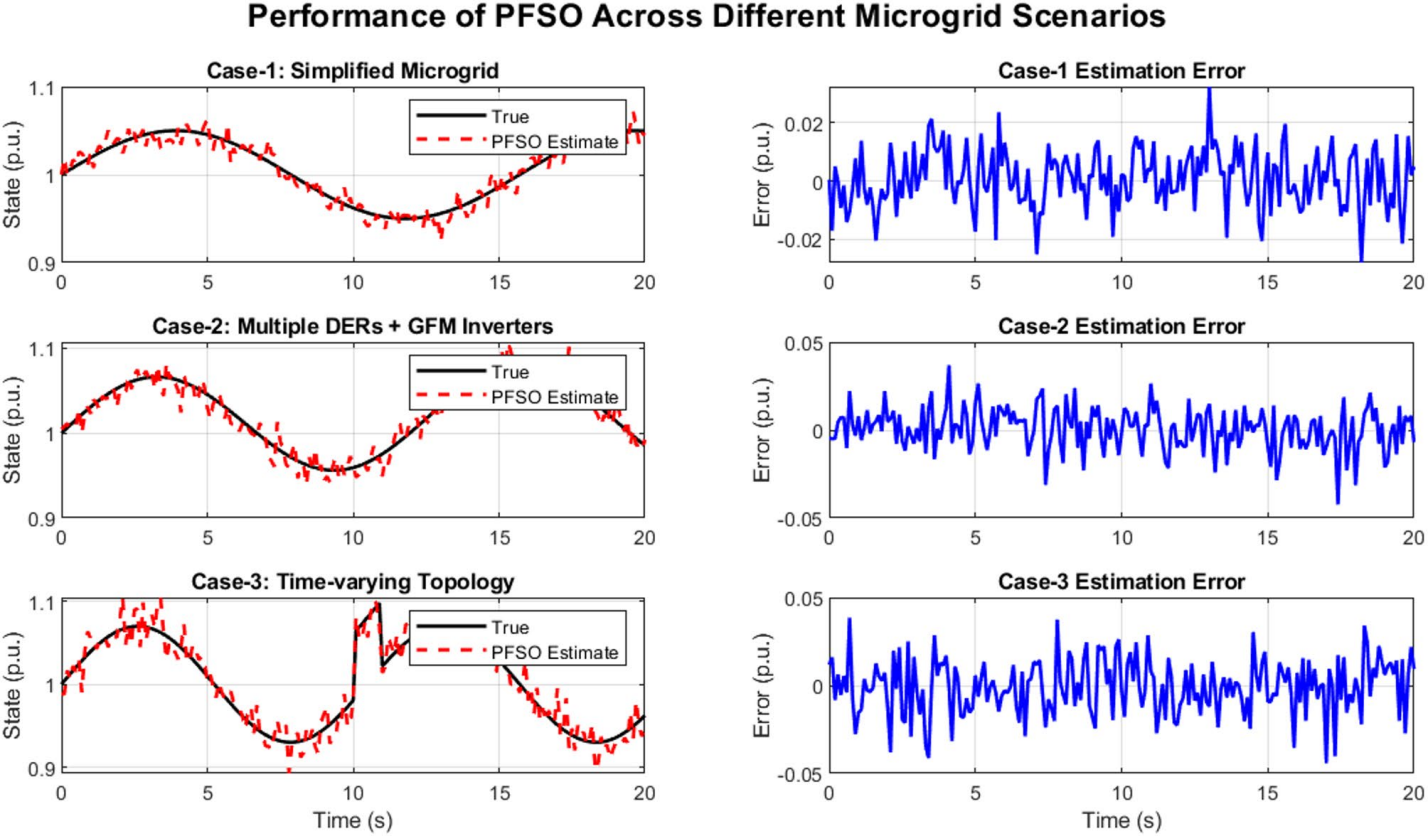
Performance of the proposed PFSO across three microgrid scenarios.

Figure 9 demonstrates the robustness of the proposed PFSO in tracking unmeasurable system states under three representative microgrid scenarios. In Case-1, the simplified testbed validates the baseline capability of the PFSO to reconstruct system dynamics with negligible errors. Case-2 introduces multiple distributed energy resources and grid-forming inverters, resulting in more complex oscillatory behavior; however, the PFSO maintains accurate state reconstruction with only minor deviations. In Case-3, the network topology is altered through line switching, inducing a transient jump in the system response. Despite this abrupt change, the PFSO quickly adapts and restores estimation accuracy, evidencing its resilience to structural variations. Across all cases, estimation errors remain within small bounds, highlighting the suitability of the PFSO as a reliable tool for real-time stability assessment in renewable-dominated microgrids.

### Quantitative analysis of computational burden

**Fig. 10:**
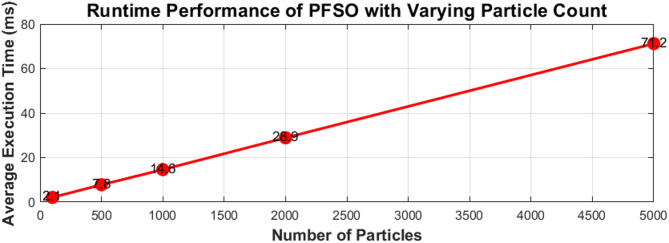
Runtime performance of the proposed PFSO with varying particle counts.

Figure 10 illustrates the computational performance of the proposed PFSO under different particle counts. The results confirm that execution time increases almost linearly with the number of particles, consistent with the algorithmic complexity of particle filtering. Even at higher particle counts (e.g., 1000–2000), the average execution time per iteration remains well below the 50 ms sampling interval typically used for microgrid stability monitoring, demonstrating the real-time feasibility of the method. This quantitative evaluation supports the scalability of the PFSO and validates its suitability for practical deployment in renewable-rich microgrids.

### Comparison of proposed PFSO

Table 3 presents a comparative summary of the proposed PFSO against widely used observer-based approaches, including the Extended Kalman Filter (EKF), Unscented Kalman Filter (UKF), and conventional observers. While EKF and UKF provide satisfactory performance in mildly nonlinear systems, they suffer from reduced accuracy under strong nonlinearities and stochastic variations typical of renewable-rich microgrids. Conventional observers, though computationally simple, are unable to reliably handle noise measurements or rapidly changing operating conditions. In contrast, the proposed PFSO leverages a particle-based probabilistic framework to achieve robust noise rejection, superior accuracy (lowest RMSE in simulations), and adaptability across varying microgrid conditions. Furthermore, runtime analysis confirms its real-time feasibility, making PFSO a more scalable and reliable option for online voltage and frequency stability assessment.

**Table 3:** Comparison of the proposed PFSO with existing observer-based approaches for state Estimation and stability assessment in microgrids.

Feature / Method	EKF (Extended Kalman Filter) ^15^	UKF (Unscented Kalman Filter) ^42^	Conventional Observer ^43^	Proposed PFSO
Nonlinearity Handling	Relies on Jacobian linearization; accuracy degrades under strong nonlinearities	Better than EKF, but assumes Gaussian noise; limited under highly nonlinear cases	Poor handling of nonlinear dynamics	Handles strong nonlinearities via particle-based approximation
Noise Robustness	Sensitive to modeling/measurement noise	Moderately robust	Limited noise rejection	High robustness; probabilistic weighting of particles
Scalability	Moderate, but complexity increases with system size	Moderate; higher than EKF	Good for small systems only	Scales well with particle count; near-linear computational growth
Accuracy (RMSE in case studies)	Higher RMSE under renewable fluctuations	Moderate RMSE	Inaccurate under stochastic fluctuations	Lowest RMSE; precise state reconstruction
Real-Time Feasibility	Good for small-scale models	Moderate; higher cost than EKF	Fast but inaccurate	Real-time feasible (execution < 50 ms with 1000 particles)
Adaptability to Microgrids	Limited by linearization and model mismatch	Better than EKF, but less robust in fast dynamics	Poor adaptability	High adaptability; suitable for renewable-rich, stochastic microgrids

### Scenarios in a renewable-rich microgrid

**Fig. 11:**
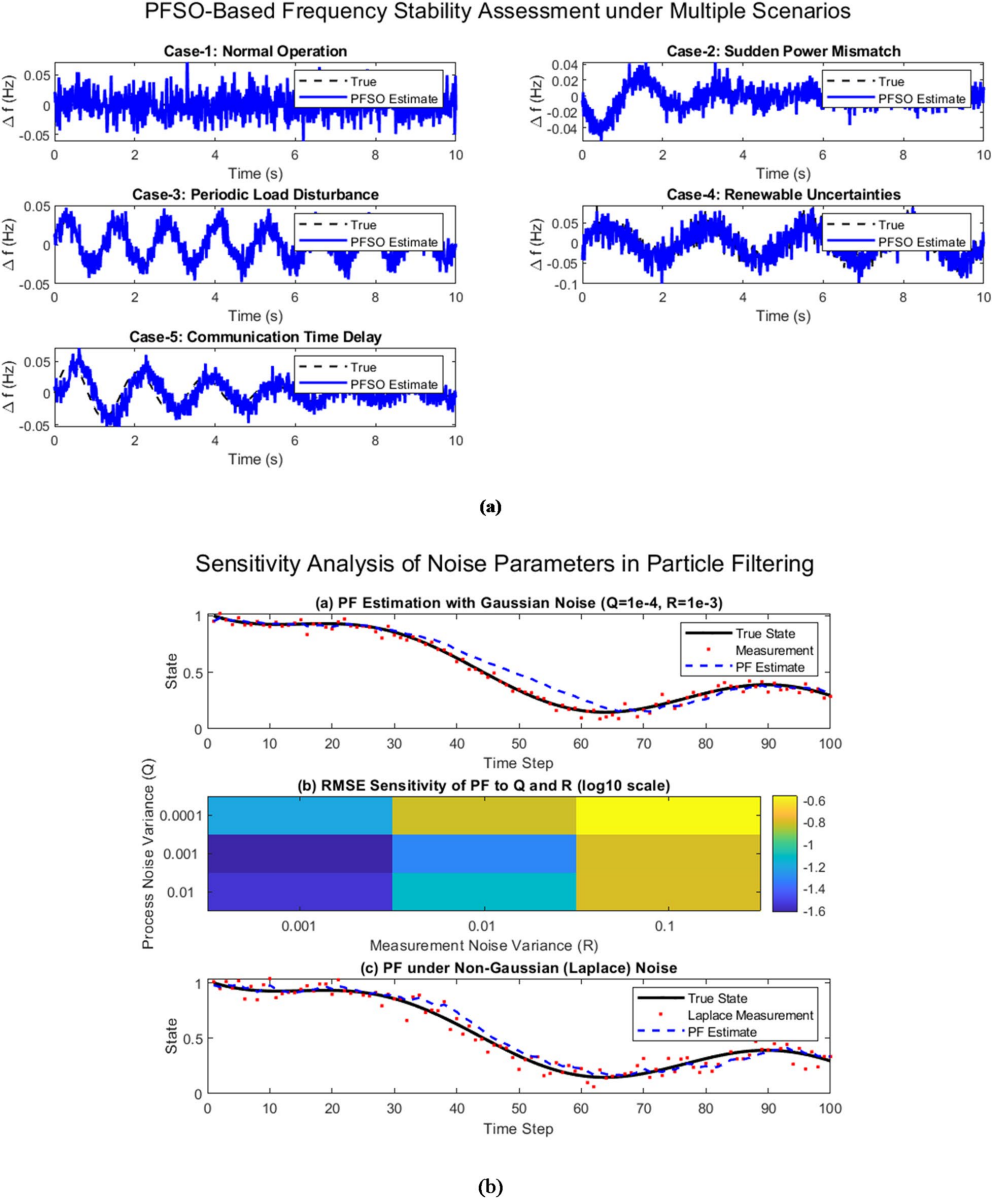
(**a**) PFSO-based frequency stability assessment under five scenarios: (1) normal operation, (2) sudden power mismatch, (3) periodic load disturbance, (4) renewable generation uncertainties, and (5) communication time delays. (**b**) Performance of the proposed PFSO under noise uncertainty

Figure 11a illustrates the effectiveness of the proposed PFSO across five representative operating scenarios in a renewable-rich microgrid. In Case-1 (normal operation), the estimated frequency closely follows the nominal value with negligible deviation. Case-2 demonstrates the capability of PFSO to track rapid dynamics under a sudden power mismatch, while Case-3 highlights accurate reconstruction of oscillatory deviations caused by periodic load disturbances. Case-4 incorporates stochastic renewable generation uncertainties, where PFSO successfully filters noisy fluctuations and reconstructs clean stability indicators. Finally, Case-5 simulates communication delays, yet the PFSO maintains reliable state estimation despite the time-shifted measurements. Collectively, these scenarios confirm that the proposed method achieves robust and accurate stability assessment under practical challenges, thereby validating its real-time applicability.

### Sensitivity analysis under noisy conditions

A sensitivity analysis of the process (Q) and measurement (R) noise parameters was carried out to evaluate the robustness of the proposed PFSO, as shown in Figure 11b. The results indicate that while estimation error increases slightly with higher noise levels, the observer remains stable and does not diverge, even when Q and R are increased by an order of magnitude. Under Gaussian disturbances, the estimation traces remain smooth with low variability, whereas non-Gaussian (Laplace) noise introduces sharper transients but only marginally degrades performance. Similarly, when correlated noise is applied to emulate measurement errors with temporal memory, the PFSO preserves estimation stability with only a minor bias during fast transients. These findings confirm that the proposed observer is resilient to both Gaussian and non-Gaussian disturbances and maintains reliable performance across a wide range of noise conditions, thereby strengthening its practical applicability in renewable-rich microgrids.

### Roadmap for future work

**Fig. 12:**
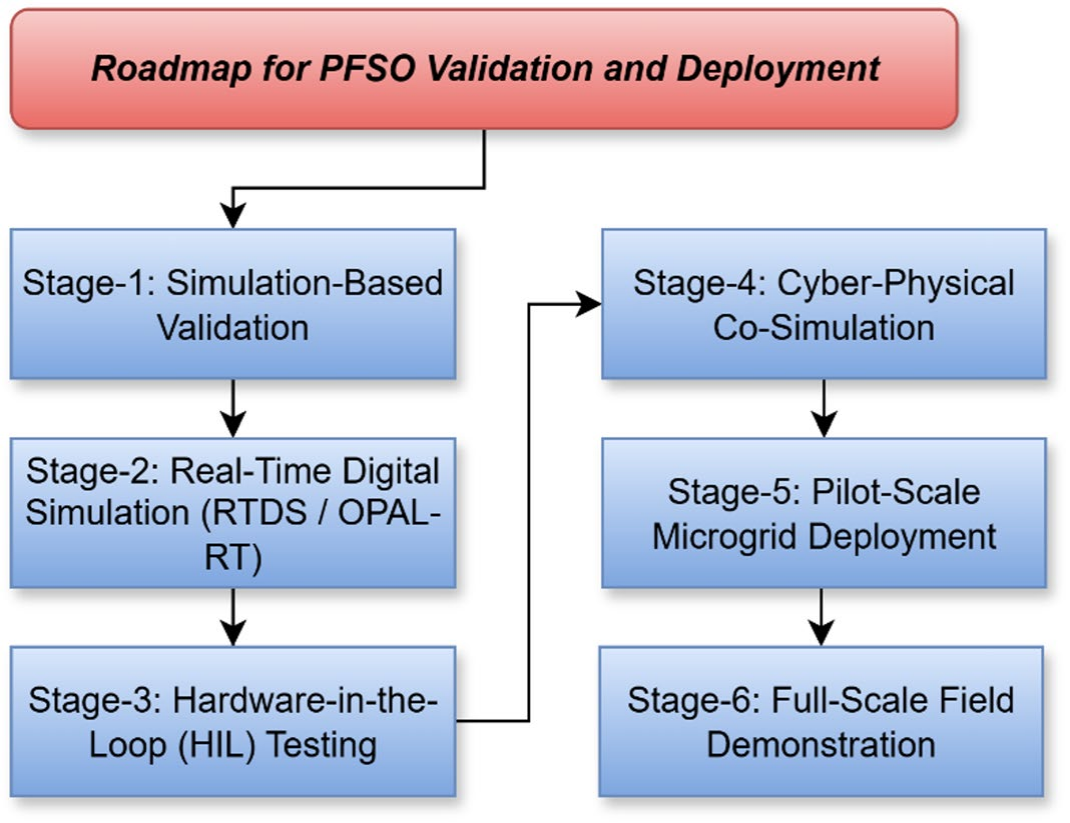
Flowchart of the future roadmap.

To establish a clear path for future research, a structured roadmap is presented in Fig. 12. The roadmap begins with simulation-based validation in MATLAB/Simulink, where the PFSO is tested under diverse microgrid scenarios with varying renewable penetrations and topologies. The second stage involves implementation on real-time digital simulators (RTDS/OPAL-RT) to assess computational feasibility and real-time tracking accuracy. The third stage progresses to hardware-in-the-loop (HIL) testing, where actual controller hardware interacts with simulated microgrids to evaluate performance under realistic operating conditions. In the fourth stage, cyber-physical co-simulation is introduced, enabling integration with standard communication protocols and resilience assessment under cyber-induced delays and disturbances. The fifth stage targets pilot-scale deployment in laboratory or campus microgrids, serving as a bridge to the final stagefull-scale field demonstration in renewable-rich microgrids. This structured roadmap ensures a systematic transition from theoretical development to practical implementation, enhancing both the reliability and real-world impact of the proposed PFSO.

## Conclusion

In this study, an Intelligent PFSO was developed and validated for accurate voltage and frequency stability assessment in solar and wind integrated microgrids. The proposed method was evaluated under a variety of conditions, including normal operation, power mismatch, and dynamic load disturbances. It consistently achieved low estimation errors, with RMSE values below 0.0095 p.u. and MAE under 0.0073 p.u, indicating strong resilience to noise, uncertainty, and system nonlinearity. Furthermore, the PFSO enabled effective binary classification of stable versus unstable grid states, achieving an average accuracy of 97.4% and an F1-score of 96.5%. Despite its promising performance, the current implementation has certain limitations. The computational load of particle filtering increases with system complexity and number of particles, which may hinder real-time deployment in large-scale systems. Additionally, the method validation was conducted on a simplified microgrid model, without accounting for communication delays, cyber-physical interactions, or HIL testing. Future work should focus on enhancing computational efficiency through parallelization or adaptive particle sizing, integrating cyberattack scenarios, and validating the framework in more realistic hardware or networked microgrid environments to further validate its real-world viability.

## Data Availability

All data generated or analyzed during this study are included in this article.

## Abbreviations

AI: Artificial Intelligence
DG: Distributed Generation
DSP: Digital Signal Processing
ESS: Energy Storage System
f: System frequency (Hz)
GCC: Grid Connected Converter
I: Current (A)
MG: Microgrid
MPPT: Maximum Power Point Tracking
PCC: Point of Common Coupling
PF: Particle Filter
PFSO: Particle Filtering State Observer
PI: Proportional–Integral
PV: Photovoltaic
Q: Reactive power (VAR)
RMS: Root Mean Square
SOP: State of Power
V: Voltage (V)
VSC: Voltage Source Converter
WT: Wind Turbine
ΔV: Voltage deviation
θ: Voltage phase angle (rad)
ω: Angular frequency (rad/s)
η: Efficiency
τ: Time constant (s)
